# The tomato 2-oxoglutarate-dependent dioxygenase gene *SlF3HL* is critical for chilling stress tolerance

**DOI:** 10.1038/s41438-019-0127-5

**Published:** 2019-04-06

**Authors:** Tixu Hu, Yuqin Wang, Qiqi Wang, Ningning Dang, Ling Wang, Chaochao Liu, Jianhua Zhu, Xiangqiang Zhan

**Affiliations:** 1 State Key Laboratory of Crop Stress Biology for Arid Areas and College of Horticulture, Northwest A&F University, No. 3, Taicheng Road, 712100 Yangling, Shaanxi China; 2 School of Biotechnology, Jiangsu University of Science and Technology, Zhenjiang, Jiangsu China; 3 Department of Plant Science and Landscape Architecture, College of Agriculture and Natural Resources, University of Maryland, College Park, MD 20742 USA

**Keywords:** Abiotic, Jasmonic acid

## Abstract

Low temperature is a major stress that severely affects plant development, growth, distribution, and productivity. Here, we examined the function of a 2-oxoglutarate-dependent dioxygenase-encoding gene, *SlF3HL*, in chilling stress responses in tomato (*Solanum lycopersicum* cv. Alisa Craig [AC]). Knockdown (KD) of *SlF3HL* (through RNA interference) in tomato led to increased sensitivity to chilling stress as indicated by elevated levels of electrolyte leakage, malondialdehyde (MDA) and reactive oxygen species (ROS). In addition, the KD plants had decreased levels of proline and decreased activities of peroxisome and superoxide dismutase. The expression of four cold-responsive genes was substantially reduced in the KD plants. Furthermore, seedling growth was significantly greater in AC or *SlF3HL*-overexpression plants than in the KD plants under either normal growth conditions with methyl jasmonate (MeJA) or chilling stress conditions. SlF3HL appears to positively regulate JA accumulation and the expression of JA biosynthetic and signaling genes under chilling stress. Together, these results suggest that *SlF3HL* is a positive regulator of chilling stress tolerance and functions in the chilling stress tolerance pathways, possibly by regulating JA biosynthesis, JA signaling, and ROS levels.

## Chill stress: Gene regulator identified in tomato plants

A gene involved in regulating responses to chill stress in tomato plants may prove valuable in reducing crop damage caused by low temperatures. A significant limiting factor in growing certain crops is cold stress – for example, tomato and cucumber plants suffer chill damage and reduced productivity at low temperatures (0 to 12 °C). Xianggiang Zhan at the Northwest A&F University in Shaanxi, China, and co-workers demonstrated that the gene *SIF3HL* is a key regulator of chilling stress tolerance in tomato plants. The team generated plants with no *SIF3HL* expressed and found that they responded poorly at low temperatures, with higher levels of reactive oxygen species and decreased levels of metabolic enzymes. Expression levels of four cold-responsive genes were also reduced. Plants overexpressing *SIF3HL*, on the other hand, coped well at low temperatures.

## Introduction

Cold stress is among the main environmental stresses limiting plant geographical distribution, growth and yield^1^. Cold stress can be divided into two categories: chilling stress (0–20 °C) and freezing stress (<0 °C). Chilling-sensitive plants, such as tomato, cucumber, and sweet pepper, can suffer from cold injury and reduced productivity when exposed to low temperature (0–12 °C). Enhancing the low temperature tolerance of chilling-sensitive plants is thus a major target for plant breeders^2^. Cold acclimation enables many temperate plants to obtain chilling tolerance after being exposed to low temperatures^3^. Cold acclimation involves many physiological and biochemical processes, including changes in membrane stability^4^, calcium fluxes^5^, and changes in cell wall properties^6^. These changes are related to the transcript levels of a number of cold-regulated genes in plants^7^.

Many cold-induced pathways are genetically activated to protect plants from cold stress. To date, most studies on this topic have been focused on the Inducer of CBF Expression 1 (ICE1-CBF) C-Repeat-Binding Factors (COR) (cold regulated) signaling pathway^8,9^. The function of the ICE1-CBF-COR pathway appears to be conserved among plant species^10,11^. It comprises the core components ICE1, CBF transcription factors, and diverse COR proteins, which are generally inducible by cold stress.

CBF proteins/transcription factors belong to the AP2/ERF (APETALA2/Ethylene-Responsive Factor)-type family of transcription factors. These CBF proteins/transcription factors can bind to promoters containing C-repeat/dehydration-responsive element (DRE) (G/ACCGAC) and regulate the transcription of downstream *COR* genes under low temperature^12–14^. Three *CBF* genes in Arabidopsis are transiently induced by cold stress and rapidly reach their maximal levels of expression under this stressor^15,16^. Ectopic expression of the tomato *SlCBF1* in Arabidopsis has been shown to increase freezing tolerance^17^. The expression of *SlCBF1* and *SlCBF2* is induced after 3 h at 10 °C during cold acclimation in tomato^18^. *AtICE1* encodes an MYC-type basic helix-loop-helix transcription factor that binds to the cis-element CANNTG in the *AtCBF3* promoter and activates *AtCBF3* expression under chilling stress^19,20^. As a homolog of AtICE1, AtICE2 activates *AtCBF1* expression during chilling stress^21^. *SlICE1* can enhance chilling tolerance and increase the expression of *SlCBF1* and *SlDRCi7* during chilling stress in tomato^22^. These data suggest that ICEs play crucial roles in the plant response to chilling stress. In addition, *ICE* is regulated negatively or positively by many other genes, such as *HOS1*, *OST1*, and *MAPKs*. *HOS1* encodes a RING finger ubiquitin E3 ligase that interacts with ICE1 and promotes ICE1 degradation by the ubiquitination/proteasome pathway^23,24^. *OST1* encodes a Ser/Thr protein kinase that positively regulates ICE1 protein stability by phosphorylating ICE1 at Ser278^25,26^. MAPK3 and MAPK6 can phosphorylate ICE1, leading to its degradation^27^.

Phytohormones involved in tolerance to abiotic stresses include abscisic acid (ABA), which is important in regulating plant stress signaling^28,29^. Several reports show that jasmonate (JA) is also important in abiotic stress responses^30^. JA is a class of fatty acid-derived phytohormone. The first step of JA biosynthesis entails the oxygenation of linolenic acid and involves many JA biosynthetic enzymes, including LOX (13-lipoxygenase), allene oxide synthase (AOS), allene oxide cyclase (AOC), and OPR [(9S,13S)-12-oxo-phytodienoic acid reductase]^31^. JA content in *ZmLOX8* mutant leaves is reduced in response to mechanical wounding^32^. JA is involved in a series of physiological and stress-related processes, such as stomatal closure and the detoxification activity of antioxidant enzymes, which can enhance plant tolerance to environmental stresses^33,34^. JAZ (JA ZIM-domain) proteins belong to the TIFY family and are important for the regulation of JA signaling. In the absence of bioactive JA, JAZs suppress the transcript levels of downstream genes, including MYC2. Under increased levels of JA, the F-box protein COI1 (CORNATINE INSENSITIVE1) interacts with JAZ proteins and promotes JAZ protein degradation through the ubiquitin degradation pathway^35,36^. JAZ proteins interact with MYB21 and MYB24, which regulate downstream gene expression^37^. JAZ1 and JAZ4 proteins interact with ICE1, which can regulate the transcription of downstream genes and influence freezing tolerance in Arabidopsis^38^. Overexpression of *OsbHLH148* increases drought stress tolerance in rice through OsbHLH148-OsJAZ-OsCOI1-JA signaling^39^. Suppression of *OsJAZ9* in rice reduces salt stress tolerance by regulating the JA signaling pathways^40^. The findings of one study suggested that the level of endogenous JA was increased under chilling stress in rice^41^. In addition, previous studies have shown that JA acts as a key signal in the ICE-CBF pathway that increases cold tolerance in Arabidopsis. Exogenous JA was found to significantly improve freezing tolerance in Arabidopsis, and the suppression of JA biosynthesis and signaling increased Arabidopsis sensitivity to freezing stress^38^. Furthermore, JA has been shown to activate the CBF pathway and increase chilling stress tolerance in tomato^42^.

Members of the 2-oxoglutarate Fe(II)-dependent oxygenase (2-OG oxygenase) superfamily, which is the second largest enzyme family, are involved in various metabolic processes, such as plant hormone biosynthesis^43^. One member of this family is involved in the biosynthesis of gibberellins^44^. *AtDMR6* (Arabidopsis downy mildew-resistant 6) belongs to the 2-OG oxygenase superfamily; its expression is induced by pathogen attack and salicylic acid (SA) treatment^45^. AtDMR6 catalyzes the hydrolysis of SA to 2,5-dihydroxybenzoic acid^46^.

In the present study, we isolated a tomato gene, *SlF3HL*, belonging to the 2-OG oxygenase superfamily that contains a 2OG-FeII-Oxy domain. *SlF3HL*, which has its highest sequence homology with *AtDMR6*, is responsive to chilling stress. Tomato plants with reduced expression of *SlF3HL* (achieved via knockdown through RNA interference) were found to be hypersensitive to chilling stress. We showed that *SlF3HL* positively regulates chilling stress tolerance, possibly by modulating JA biosynthesis, the expression of JA signaling molecules (such JAZ proteins) and the accumulation of reactive oxygen species (ROS) under chilling stress conditions.

## Materials and methods

### Growth conditions and analysis of chilling stress tolerance in tomato

Tomato (*Solanum lycopersicum* cv. Alisa Craig, AC) seeds of AC and *SlF3HL* transgenic lines were sown in black plastic pots containing soil, perlite, and vermiculite (v/v/v = 2:1:1). The seedlings were grown under normal conditions (25 °C, 60–65% relative humidity, 16-h day/8-h night). One-month-old tomato plants were used in the subsequent experiments. For the chilling tolerance assays, plants were transferred to a growth chamber at 4 °C and maintained under the same relative humidity and photoperiod as during their previous growth. For treatment with MeJA and DIECA (JA biosynthesis inhibitor sodium diethyldithiocarbamate trihydrate), seeds of AC and *SlF3HL* transgenic lines were surface-sterilized and sown on 1/2 MS (Murashige-Skoog) medium plates for germination under normal conditions (25 °C, 60–65% relative humidity, 16-h day/8-h night). After 6 days, seedlings of similar size were transferred to 1/2 MS medium plates supplemented with 0 or 5 μM MeJA or with 0 or 10 μM DIECA and placed in a 4 °C growth chamber. Other seedlings were treated in the same manner but were grown at 25 °C in a separate growth chamber. After 7 days of these treatments, the fresh weights, root lengths, and hypocotyl lengths of the seedlings were measured.

### RNA extraction and quantitative real-time PCR (qPCR) analysis

Total RNA was extracted from various tissues of the tomato plants using Trizol (Invitrogen, USA). cDNA was synthesized from total RNA using a cDNA synthesis kit (Toyobo Bio-Technology, China) following the manufacturer’s instructions. qPCR was carried out on a Bio-Rad CFX96^TM^ instrument (Bio-Rad, USA) following the manufacturer’s instructions. The specific primers are listed in Table S1. The actin gene (Accession No. BT013524) was used as the internal control. The transcript level of specific genes was measured using the cycle threshold (Ct) 2^−ΔΔCt^ method^47^.

### Relative electrical conductivity (REC) assays

The REC was measured as previously described^48^. Ten leaf disks (each 0.6 cm in diameter) from each line were harvested and placed in a glass tube containing 10 ml of deionized water. After the glass tube was shaken at 100 cycles per min for 30 min under normal laboratory conditions, the initial electrical conductivity (*C*_1_) was measured with a conductivity meter (Mettler Toledo FE30, Switzerland). Then, the glass tube containing leaf segments in distilled water was boiled for 30 min. The final electrical conductivity (*C*_2_) was measured after cooling the tube to ambient temperature. The electrical conductivity (*C*_0_) of the deionized water was measured and used as the blank. REC was calculated as (*C*_1_ − *C*_0_)/(*C*_2_ − *C*_0_) × 100%.

### Malondialdehyde (MDA) content measurement

The level of MDA was determined using the thiobarbituric acid method as previously described^49^. Leaf samples (approximately 0.3 g per sample) were collected from the second leaf from the top of 1-month-old tomato plants grown under normal conditions or under chilling stress. Samples were ground with 5 ml of ice-cold 10% TCA (trichloroacetic acid). The homogenates were centrifuged at 12,000 × *g* for 10 min at 4 °C. A 2-ml volume of the supernatant and 2 ml of 10% TCA containing 0.6% thiobarbituric acid were mixed together and boiled for 15 min. After the mixture was cooled to ambient temperature, the homogenate was centrifuged for 10 min at 10,000 × *g*. Absorbance was recorded at 450, 532, and 600 nm using an Infinite M200 microplate reader (Tecan, Switzerland).

### Proline content measurement

The level of proline was determined using the acid ninhydrin method as previously described^50^. Leaf samples (approximately 0.3 g per sample) were ground with 5 ml of 3% sulfosalicylic acid and boiled for 30 min with shaking. After cooling the suspension to ambient temperature, the suspension was centrifuged for 10 min at 12,000 × *g*. A 1-ml volume of the supernatant, 1 ml of acid ninhydrin, and 1 ml of acetic acid were mixed together and boiled for 60 min. After cooling the mixture to ambient temperature, the preparation and 3 ml of toluene were mixed together and centrifuged for 10 min at 12,000 × *g*. Subsequently, 150 μl of the toluene phase was recovered, and its absorbance at 520 nm was measured with an Infinite M200 microplate reader. The level of proline was calculated using a standard curve relating proline concentration to absorbance.

### O_2_^−^ content measurement

The level of O_2_^−^ was determined as previously described^51^. Leaf samples (approximately 0.3 g per sample) were ground with 2 ml of ice and precooled 50 mM phosphate buffer solution (PBS, pH 7.8) and then centrifuged at 12,000 × *g* for 10 min at 4 °C. A 1-ml volume of the supernatant, 1 ml of 10 mM hydrochloride hydroxylamine, and 3 ml of PBS were mixed together and incubated for 20 min at 25°C. Then, the reaction mixture, 1 ml of 7 nM α-naphthylamine and 1 ml of 17 nM p-aminobenzenesulfonic acid were mixed together and incubated for 30 min at 30 °C. Finally, the absorbance at 530 nm of 200 μl of the reaction mixture was determined using an Infinite M200 microplate reader. The O_2_^−^ content was calculated using a standard curve relating O_2_^−^ concentration to absorbance.

### H_2_O_2_ detection

H_2_O_2_ was detected as previously described^52^. Leaves of plants of the AC and transgenic tomato lines were stained with 3,3′-diaminobenzidine to determine the level of H_2_O_2_ under normal and low-temperature conditions. Four-week-old plants of the AC and transgenic lines were transferred to growth chambers at 25 or 4 °C. After 5 days, leaves were soaked in 1 mg/ml DAB (3,3′-diaminobenzidine) in 50 mM Tris-base (pH 5.0). The plants were then incubated for 24 h in the dark at 25 °C. Chlorophyll was removed by incubation with 70% ethanol before H_2_O_2_ was assessed.

### Measurement of antioxidant enzyme activities

Antioxidant enzymes activities were measured as previously described^53^. In brief, leaf samples (approximately 0.2 g per sample) were ground with 2 ml of ice and added to precooled 50 mM PBS (pH 7.8) containing 1 mM EDTA (ethylenediaminetetraacetic acid), 0.1% (v/v) Triton X-100, and 1% (w/v) PVP (polyvinylpolypyrrolidone). The suspension was centrifuged at 12,000 × *g* for 10 min at 4 °C. The supernatants were then used for measurement of the activities of the antioxidant enzymes SOD (superoxide dismutase) and POD (peroxidase).

### JA content measurement

Leaf samples (approximately 0.2 g per sample) were ground in liquid nitrogen. The powdered leaf samples were resuspended in 2 ml of extraction solution containing 0.4 ml of methanol, 1.58 ml of isopropanol and 0.02 ml of glacial acetic acid. A 1.5-ml volume of the suspension was centrifuged at 12,000 × *g* for 10 min at 4 °C. The supernatant was collected and concentrated under a nitrogen stream. The concentrated samples were then redissolved in 200 μl of methanol and filtered by a 0.22-μm PTFE filter. The content of JA was analyzed by using ultrahigh-performance liquid chromatography coupled to electrospray ionization tandem spectrometry (UHPLC/ESI-MS/MS) as previously described^54^.

## Results

### Expression patterns of *SlF3HL* in tomato

**Fig. 1:**
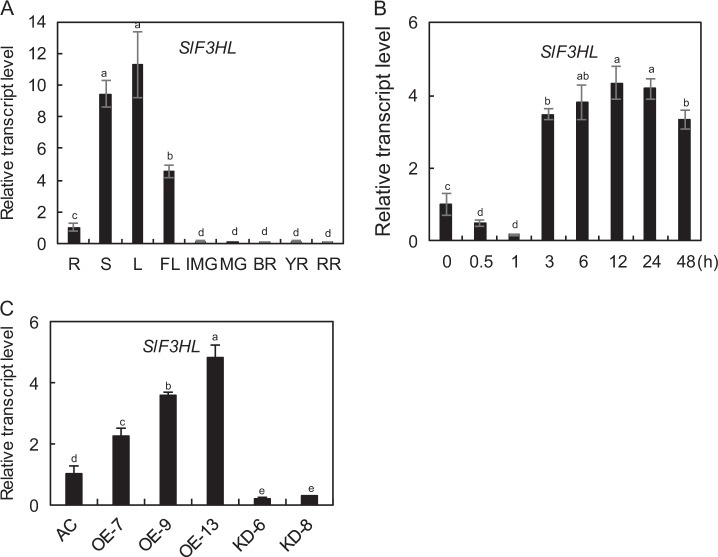
qRT-PCR analysis of transcript abundance of *SlF3HL*. **a** The transcript levels of *SlF3HL* in different tomato organs: R, Root; S, Stem; L, Leaf; FL, Flower; IMG, Immature fruit; MG, Mature green fruit; BR, Breaker stage fruit; YR, Yellow stage fruit; RR, Red ripe stage fruit; **b** The transcript abundance of *SlF3HL* in response to chilling stress at 4 °C; **c** *SlF3HL* expression in young leaves of the wild type (AC) line, three *SlF3HL*-OE (overexpression) lines, and two *SlF3HL*-KD (knockdown) lines. The data are presented as the mean ± SD (*n* = 3). Values with different letters significantly differ according to analysis of variance (ANOVA) and least significant difference (LSD) tests (*p* < 0.05)

In a previous study, *SlF3HL* (GenBank Accession No. NP_001233840 and SGN Accession No. Solyc03g080190) was identified as a gene encoding a 2-oxoglutarate-dependent dioxygenase protein (a member of the 2-OG oxygenase superfamily), which shares the highest sequence similarity with the AtDMR6 protein^55^. We examined the expression patterns of *SlF3HL* in various tomato organs using qRT-PCR analysis. As shown in Fig. 1a, the transcript levels of *SlF3HL* were higher in leaves, stems, and flowers than in roots and were at lowest at all developmental stages of fruits. We also determined the transcript abundance of *SlF3HL* in response to chilling treatment (4 °C) in tomato. The expression of *SlF3HL* was substantially decreased during the first 1 h of chilling treatment and then increased at and beyond 3 h of chilling treatment (Fig. 1b). These data suggest that *SlF3HL* may be involved in chilling stress responses in tomato.

### Generation of *SlF3HL* transgenic plants

To determine the role of *SlF3HL* in chilling stress responses in tomato, we generated 25 *SlF3HL*-overexpression (OE) transgenic tomato plants and 20 RNA interference (KD) transgenic tomato plants. Three independent OE and two independent KD transgenic lines were chosen for further analysis. The expression of *SlF3HL* was substantially higher in the OE lines and lower in the KD lines than in AC (Fig. 1c). The transcript levels of *SlF3HL* in leaves of the OE-7, OE-9, and OE-13 lines were 2.2-fold, 3.5-fold, and 4.8-fold higher than those in the AC leaves, whereas *SlF3HL* transcript levels were 0.2-fold lower in the KD-6 leaves and 0.3-fold lower in KD-8 leaves than in the AC leaves (Fig. 1c).

### *SlF3HL* is important for chilling stress tolerance

**Fig. 2:**
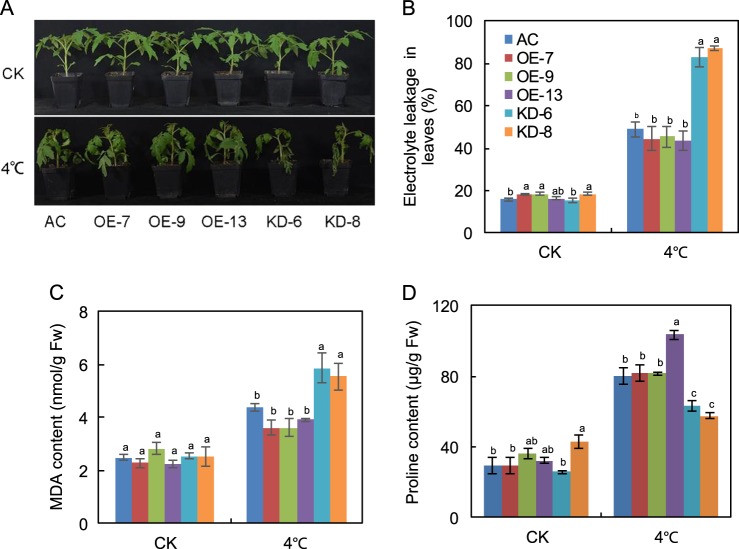
*SlF3HL* is important for chilling stress tolerance. **a** The phenotypes of plants of the AC and *SlF3HL* transgenic lines treated with or without chilling stress for 5 days; **b** Chilling tolerance of plants shown in A as determined by leaf electrolyte leakage assays; **c** MDA content in leaves of plants shown in **a**. **d** Proline content in leaves of plants shown in **a** CK, normal growth conditions. The data are presented as the mean ± SD (*n* ≥ 5). Values with different letters under normal or chilling stress conditions significantly differ according to ANOVA and LSD tests (*p* < 0.05)

To determine whether alteration of *SlF3HL* expression affects chilling stress tolerance, we treated 5-week-old transgenic and wild-type plants at 4 °C for 7 days. Following this chilling stress, leaves were severely wilted on the KD plants but only slightly wilted on the OE and AC plants (Fig. 2a), showing that a reduction in the transcript level of *SlF3HL* increased plant sensitivity to chilling stress. Levels of MDA, proline, and ion leakage are indicators of membrane damage caused by chilling stress. Although the content of MDA increased in all of the plants after chilling stress, MDA levels were significantly higher in the KD plants than in the AC and OE plants (Fig. 2c). Similarly, the electrolyte leakage levels were significantly higher in the KD plants than in the AC and OE plants (Fig. 2b). In contrast, proline content was significantly lower in the KD plants than in the AC and OE plants (Fig. 2d). These data indicate that *SlF3HL* is critical for chilling stress tolerance in tomato plants.

### *SlF3HL* is important for ROS detoxification under chilling stress

**Fig. 3:**
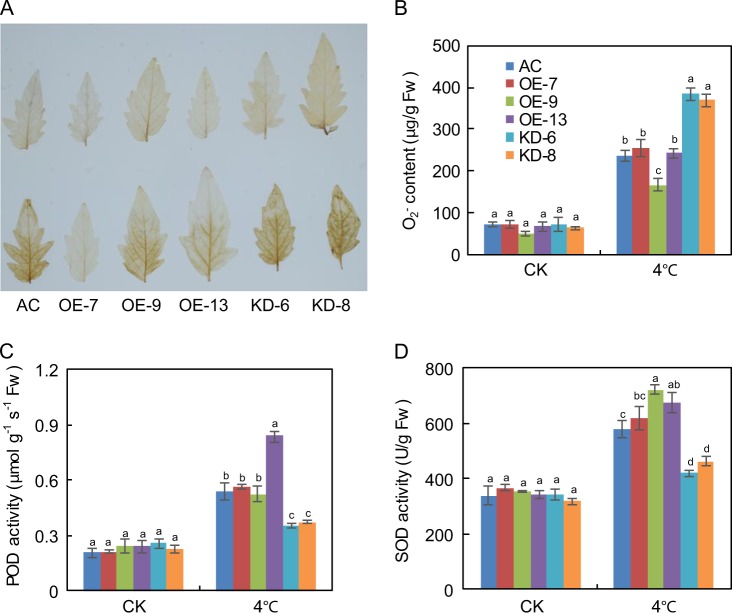
*SlF3HL* is important for ROS scavenging under chilling stress. **a** 3,3′-diaminobenzidine (DAB) staining for hydrogen peroxide (H_2_O_2_) in leaves from plants under normal and chilling stress conditions; **b** Levels of superoxide radical (O_2_^−^) in leaves from plants treated with or without chilling stress; **c** POD activity in AC and *SlF3HL* transgenic lines under normal and chilling stress conditions; **d** SOD activity in AC and *SlF3HL* transgenic lines under normal and chilling stress conditions. The data are presented as the mean ± SD (*n* = 5). Values with different letters under normal or chilling stress conditions significantly differ according to ANOVA and LSD tests (*p* < 0.05)

Plants often produce large quantities of ROS upon exposure to low temperatures. To investigate whether altered redox status contributes to increased cold sensitivity in *SlF3HL* KD plants under chilling stress, we measured the accumulation of H_2_O_2_ using DAB staining^52^. All transgenic and AC plants accumulated basal levels of H_2_O_2_ under normal growth conditions. After chilling stress, OE plants accumulated much lower levels of H_2_O_2_ than did KD plants (Fig. 3a). We also determined the levels of superoxide radicals (O_2_^−^) in AC and transgenic plants under chilling stress. Without chilling stress, there was no significant difference in O_2_^−^ content between the AC and transgenic plants. However, with chilling stress, the KD plants accumulated higher levels of O_2_^−^ than did the AC and OE plants (Fig. 3b). The accumulation of excessive ROS is the major factor leading to cell damage under abiotic stresses. Because plant cells can scavenge excessive ROS using a complex antioxidant system that includes POD and SOD, we assessed POD and SOD activities in the AC and transgenic plants under chilling stress. POD and SOD activities did not differ between AC and transgenic plants under normal growth conditions (Fig. 3c, d). When the plants were exposed to 4 °C for 48 h, POD and SOD activities increased in all of the plants but were much higher in the AC and OE plants than in the KD plants (Fig. 3c, d). These data indicate that altered redox status may contribute to the increased sensitivity to chilling stress in KD plants.

### *SlF3HL* positively regulates cold-responsive gene expression in tomato

**Fig. 4:**
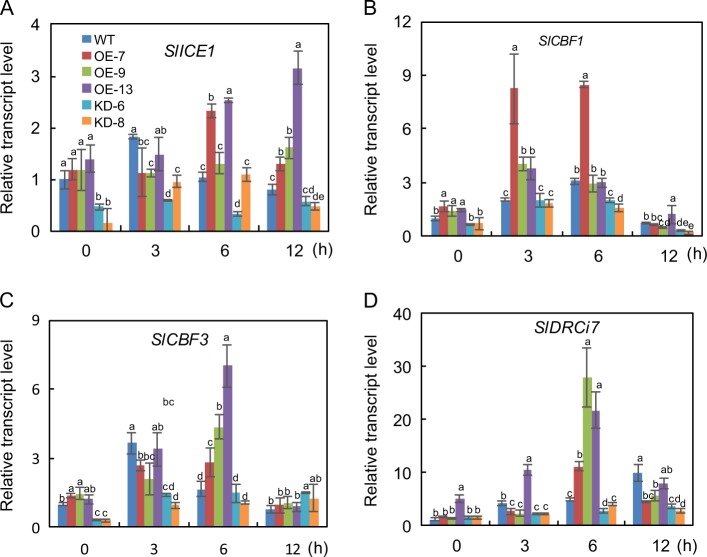
Expression of four cold-responsive genes in AC and *SlF3HL* transgenic lines under normal and chilling stress conditions. The data are presented as the mean ± SD (*n* = 3). Values with different letters at a particular time point of chilling stress significantly differ according to ANOVA and LSD tests (*p* < 0.05)

To determine whether *SlF3HL* regulates chilling stress through the ICE-CBF pathway, we measured the transcript abundances of the ICE-CBF pathway-related genes in AC and transgenic plants under chilling stress. The tomato gene *SlCBF1* can be induced by chilling stress and can increase freezing tolerance in Arabidopsis^17^. Furthermore, overexpression of *SlICE1* has been shown to enhance chilling stress tolerance in tomato^22^. The expression of *SlICE1*, *SlCBF1*, *SlCBF3*, and the *SlCBF1*-dependent gene *SlDRCi7* was investigated in the AC and transgenic plants during chilling stress (Fig. 4). The expression levels of *SlICE1*, *SlCBF1*, and *SlCBF3* were slightly higher in the OE plants than in the KD plants under normal conditions, but expression of *SlDRCi7* did not significantly differ among the AC, OE, and KD plants under these conditions. Although the expression of these genes was upregulated in all plants after chilling stress treatment, the transcript levels of these genes were substantially higher in the AC and OE plants than in the KD plants (Fig. 4). These results suggest that *SlF3HL* may be involved in chilling tolerance regulation in tomato plants via the ICE-CBF pathway.

### SlF3HL regulates JA biosynthesis and controls the expression of JA biosynthesis genes and downstream JA signaling genes

**Fig. 5:**
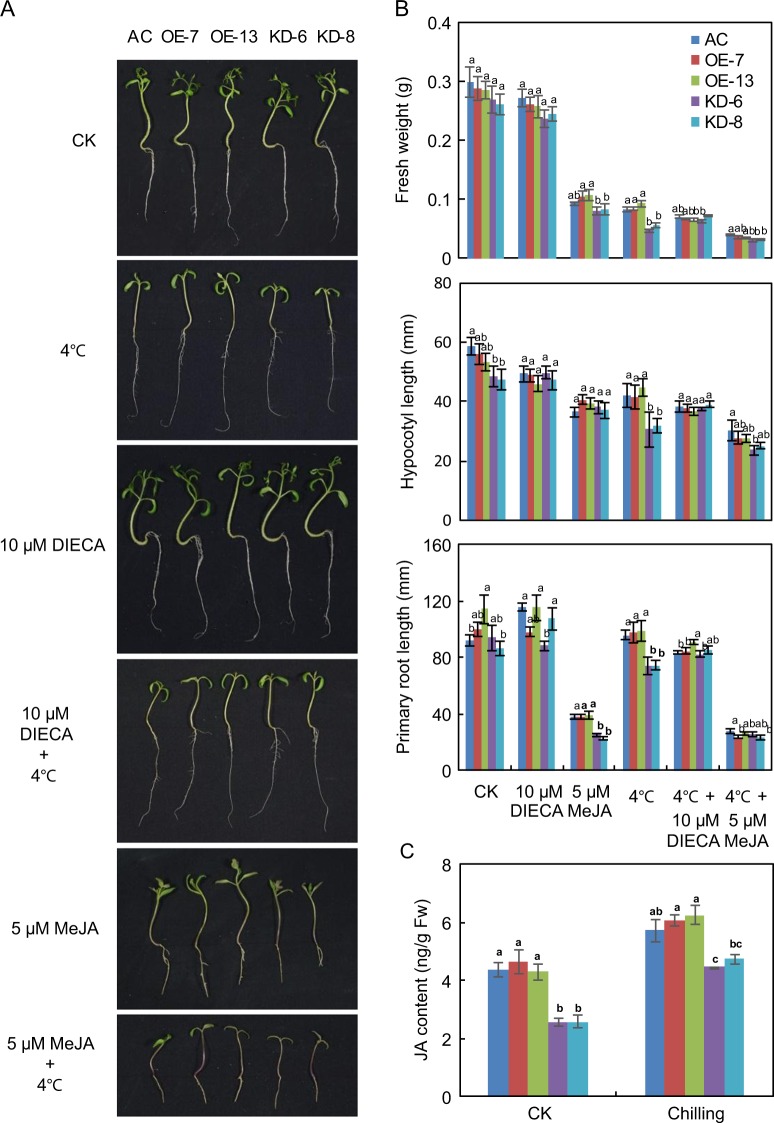
*SlF3HL* is important for jasmonate biosynthesis under chilling stress. **a** Phenotypes of plants of AC and *SlF3HL* transgenic lines under normal and chilling stress conditions with or without MeJA or DIECA; **b** Fresh weights, primary root lengths, and hypocotyl lengths of plants shown in **a**. **c** Jasmonate levels in transgenic lines. The data are presented as the mean ± SD (*n* ≥ 3). Values with different letters under a particular growth condition significantly differ according to ANOVA and LSD tests (*p* < 0.05)

JA plays key roles in regulating plant responses to various environmental stresses^56^. To determine whether *SlF3HL* is involved in JA-regulated chilling stress responses in tomato, we sowed seeds of AC-generation and T_2_-generation transgenic plants on 1/2 MS medium or 1/2 MS medium containing 5 μM MeJA or 10 μM DIECA. The germinated seedlings were then grown for an additional 10 days with or without chilling stress. The growth of AC and transgenic seedlings was significantly reduced under chilling stress. However, the fresh weights, root lengths, and hypocotyl lengths of the AC and OE plants were significantly greater than those of the KD plants under chilling stress (Fig. 5). Fresh weight and primary root length were significantly greater in the AC and OE plants than in the KD plants under normal conditions with or without exogenous MeJA. Fresh weight and primary root length did not significantly differ among the AC, OE, and KD plants grown under normal conditions with DIECA or under chilling stress conditions with DIECA or MeJA (Fig. 5a, b). These data suggest that knockdown of *SlF3HL* in tomato increases sensitivity to the JA signal. Chilling stress induced the accumulation of JA, and the levels of JA were significantly lower in the KD plants than in the AC and OE plants under both normal and chilling stress conditions (Fig. 5c). These data indicate that SlF3HL regulates the level of JA accumulation under normal and chilling stress conditions.

**Fig. 6:**
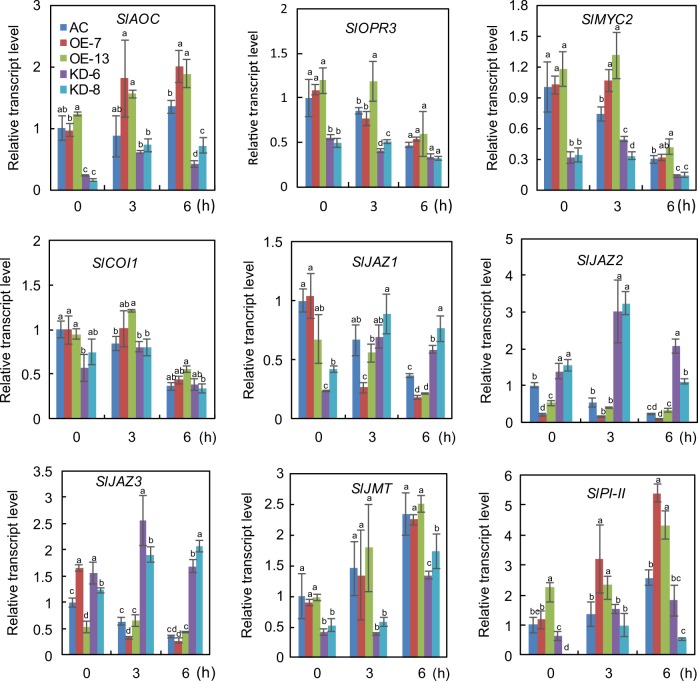
Expression of JA biosynthesis and signal transduction-related genes in AC and *SlF3HL* transgenic lines under chilling stress. The data are presented as the mean ± SD (*n* = 3). Values with different letters at a particular time point of chilling stress significantly differ according to ANOVA and LSD tests (*p* < 0.05)

We performed qRT-PCR analysis to determine whether the transcript levels of JA biosynthesis and signaling pathway-related genes are altered in *SlF3HL* KD plants under chilling stress. The transcript levels of *SlAOC* and *SlOPR3*, which are involved in the biosynthesis of JA, were substantially lower in the KD plants than in the AC and OE plants under both normal conditions and chilling stress conditions (Fig. 6). We also examined the transcript levels of JA signaling pathway genes (*SlJAZs*, *SlMYC2*, and *SlCOI1*) in the AC and *SlF3HL* transgenic plants. The expression of *SlMYC2* was higher in the AC and OE plants than in the KD plants under both normal and chilling stress conditions (Fig. 6). The expression of *SlCOI1* was slightly lower in the KD plants than in the AC and OE plants under normal conditions but was similar among all genotypes tested under chilling stress conditions (Fig. 6). All of the tested *SlJAZs* except *SlJAZ1* are cold inducible. The transcript levels of *SlJAZ1* were lower in the KD plants than in the AC and OE plants under normal conditions but were increased in the KD plants under chilling stress conditions (Fig. 6). In contrast, the expression levels of *SlJAZ13* and *SlJAZ2* were considerably higher in the KD plants than in the AC and OE plants under both normal and chilling stress conditions (Fig. 6). The two JA-responsive genes, *SlPI-II* and *SlJMT*, are cold inducible, but their expression levels were much lower in the KD plants than in the AC and OE plants (Fig. 6). Together, these data suggest that *SlF3HL* is involved in regulating JA accumulation under chilling stress and that it may function by altering the expression of JA biosynthetic and signaling-related genes.

## Discussion

In this study, we found that the tomato gene *SlF3HL* contributes to chilling stress tolerance. SlF3HL belongs to the 2-OG oxygenase family, and the members of this family are involved in various biological processes including plant hormone biosynthesis^43^. SlF3HL has highest sequence similarity with its Arabidopsis homolog, AtDMR6^55^. AtDMR6 catalyzes salicylic acid to produce 2,5-dihydroxybenzoic acid, which affects leaf senescence and the defense against *Pseudomonas syringae* pv *tomato* DC3000^46^. We hypothesized that SlF3HL functions in cold stress responses because it is cold inducible. To determine the role of *SlF3HL* in cold stress responses, we produced loss-of-function and potential gain-of-function transgenic tomato plants. The *SlF3HL* knockdown plants created through RNA interference were hypersensitive to chilling stress, suggesting that *SlF3HL* is a positive regulator of chilling stress tolerance.

Many studies have reported that ROS production is triggered when plants are exposed to abiotic stresses^57^. Chilling stress can lead to the production of large amounts of ROS, which in turn can cause oxidative damage to DNA, proteins and membrane lipids^58–60^. In the present study, we found that H_2_O_2_ and O_2_^−^ levels were greater in the *SlF3HL*-KD plants than in the AC and *SlF3HL*-OE plants under chilling stress (Fig. 3a, b). The levels of MDA and ion leakage, which reflect the degree of cellular membrane damage, were significantly higher in the *SlF3HL*-KD plants than in the AC and OE plants (Fig. 2b, c). Proline is a small compatible molecule that can protect plants from abiotic stresses, including oxidative stress. Our results showed that proline content was lower in the KD plants than in the AC and OE plants under chilling stress (Fig. 2d). It is possible that the increased sensitivity of *SlF3HL*-KD plants to chilling stress is at least partially caused by the alterations in these physiological parameters, i.e., by increases in ROS and MDA levels and by reductions in proline levels.

Plants have evolved complex mechanisms that involve both nonenzymatic and enzymatic antioxidants and that work in concert to scavenge overproduced ROS and protect cells during abiotic stress^60,61^. The enzymatic antioxidant system comprises ascorbate peroxidase, catalase, SOD, and POD, which scavenge ROS^62^. We found that the activities of SOD and POD were substantially lower in the KD plants than in the AC and OE plants during chilling stress (Fig. 3c, d). These data suggest that *SlF3HL* is important for the detoxification of ROS under chilling stress in tomato plants.

The plant hormone JA modulates many physiological processes, such as root initiation, leaf senescence, and biotic and abiotic stress responses^32,63–65^. Previous studies have demonstrated that exogenous JA can inhibit various aspects of seedling growth, including root growth and hypocotyl elongation^66,67^. In the present study, the growth of AC and *SlF3HL* transgenic seedlings did not significantly differ in the absence of MeJA treatment under normal conditions, but growth inhibition by MeJA treatment under normal conditions was greater for the *SlF3HL*-KD seedlings than for the AC and OE seedlings (Fig. 5). These results suggest that *SlF3HL*-KD plants may be sensitive to JA signaling. Although growth was greater for AC and OE seedlings than for KD seedlings during chilling stress, growth did not significantly differ among these seedlings during chilling stress when the medium was supplemented with MeJA or the JA biosynthesis inhibitor DIECA (Fig. 5). These data suggest that *SlF3HL* controls chilling stress responses, possibly through the JA signaling pathway.

*AOC* and *OPR3* are key enzymes in JA biosynthesis. The content of JA-Ile was shown to be significantly decreased in the *opr3-3* mutant relative to wild type in tomato^68^. In other work, RNAi interference of *SlOPR3* failed to result in accumulated JA after wounding^69^. The present finding that the *SlF3HL*-KD plants produced reduced levels of *SlAOC* and *SlOPR3* transcripts relative to the levels in AC and OE plants during chilling stress (Fig. 6) suggests that *SlF3HL* is a positive regulator of JA biosynthesis. JAZ proteins are key transcriptional repressors of the jasmonate-sensing and jasmonate-signaling pathway and play key roles in abiotic stress responses^70^. COI1, JAZs, and MYC2 are involved in JA signaling transduction and regulate JA-induced abiotic stress responses^40,71,72^. In the current study, the growth of AC and *SlF3HL*-OE tomato seedlings was significantly greater than that of *SlF3HL* KD plants under normal conditions with MeJA treatment. The transcript levels of *SlMYC2* in *SlF3HL* were significantly lower in the KD plants than in the AC and OE plants (Fig. 6). In contrast, the expression of *SlJAZs* was generally higher in the *SlF3HL*-KD plants than in the AC and OE plants under chilling stress (Fig. 6). These results suggest that *SlF3HL* may control chilling stress tolerance in a JA-dependent manner.

**Fig. 7:**
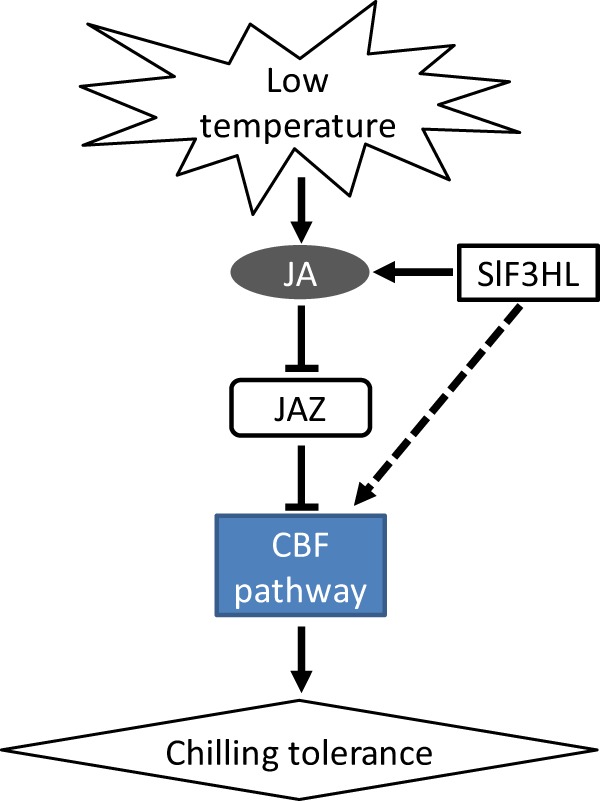
A working model of how SlF3HL functions under cold stress. Cold stress induces jasmonate biosynthesis, and jasmonate represses the transcription of JAZ genes. JAZ proteins are generally negative regulators of chilling stress tolerance. SlF3HL positively regulates JA biosynthesis and its downstream signaling

Based on our results and those of previous studies of model plant species, we propose a working model of SlF3HL-regulated JA biosynthesis and downstream signaling under chilling stress (Fig. 7): *SlF3HL* positively regulates the level of JA under chilling stress, and JA negatively regulates the transcript levels of *JAZs*, resulting in enhanced chilling stress tolerance. *SlF3HL* also positively affects the expression of cold-responsive genes.

## Supplementary information

Supplementary TableS1
