# Supplementary material for: The tomato 2-oxoglutarate-dependent dioxygenase gene SlF3HL is critical for chilling stress tolerance
Source: Hortic Res. 2019 Apr 6;6:45. doi: 10.1038/s41438-019-0127-5 (PMC6441657; doi:10.1038/s41438-019-0127-5)
Supplement: Supplementary file 1 — Supplementary TableS1 [file 41438_2019_127_MOESM1_ESM.pdf]

Table S1. List of primers used in this study.

Gene	ID	Forward primer (5'-3')	Reverse primer (5'-3')
SlF3HL (OE)	Solyc03g080190	GGGGACAAGTTTGTACAAAAAAGCAGGCTGATTCCATGGAAACCAAAGT	GGGGACCACTTTGTACAAGAAAGCTGGGTATCCCTATGAACCAAACGAC
SlF3HL (RNAi)	Solyc03g080190	GGGGACAAGTTTGTACAAAAAAGCAGGCTCGAATCCGATAGACCACGTC	GGGGACCACTTTGTACAAGAAAGCTGGGTTTGATAATCTCATGGTCTTTGAAGG
qSlCOI1	Solyc05g052620	AACGTGAGATGGATGCTTCTGGGA	ACAGCAGCCTCTCACTTCTAGCTT
qSlMYC2	Solyc08g076930	AATTTGGGGCTGGGGAGGAGAAT	CTCCACCTGACTTCATGCCCGAA
qSlJAZ2	Solyc12g009220	GCAAGGTGACCGGACAGAAATCTC	TGTGGTGGTAGTAGCTGTTTGTTGA
qSlJAZ1	Solyc07g042170	CGCAATTTCGATTCAGCTGGTTCT	CGACTCAGCCTTCATTCCACCTTG
qSlJAZ3	Solyc03g122190	CCTCCAGATTAAGCCAGACTCTTGC	CTGGATTTGCTTGTGTTGCTCCTG
qSlPI-II	Solyc03g020080	ACCCAAGGCAAATATGTACCCTGT	TAACACACAACTTGATGCCCACA
qSlJMT	Solyc09g091550	GGTTCAAAGTGCATGAGAGCT	TACACCACACACTGAAGGAAA
qSlAOC	Solyc02g085730	CAGCAGGACTCTGCATTCTG	CGGTGACGGCTAGGTAAGTT
qSlOPR3	Solyc07g007870	ATGTTGGTCGTGCATCTCAT	GGTTCCAATTGCTCTTGGTT
qSlCBF1	Solyc03g026280	TTCATCGTCATCGTCGTTTTCT	TCCTCTTCCTGATTCCCCTGT
qSlDRCi7	Solyc04g082200	TTGTGTTTCTGTGTTGTTTTGG	GCACATACATATGCACTTACATACAG
qSlCBF3	Solyc03g026270	TGCCGGGTTTACTTACGAAT	TCAGCTTCCACATGATCTCC
qSlF3HL	Solyc03g080190	CAGGCAGTAAGTAACGGTAAGTAC	GAGATCCATCTTCTGTCAGCAG
qactin	BT013524	GTCCTCTTCCAGCCATCCA	ACCACTGAGCACAATGTTACCG
